# A Rare Case of Mollaret’s Meningitis Complicated by Chronic Intractable Migraine and Papilledema: Case Report and Review of Literature

**DOI:** 10.7759/cureus.7026

**Published:** 2020-02-18

**Authors:** Kinjal P Gadhiya, Vinod Nookala

**Affiliations:** 1 Internal Medicine, University of Pittsburgh Medical Center (UPMC) Pinnacle, Harrisburg, USA

**Keywords:** mollaret, meningitis, migraine

## Abstract

Mollaret’s meningitis is characterized by recurrent episodes of aseptic meningitis that last two to seven days and resolve spontaneously without any residual neurological deficit or complication. Viruses are the most common cause of aseptic meningitis and herpes simplex virus (HSV) type 2 has been noted as the most commonly associated virus in Mollaret’s meningitis. We describe a rare case of a female who had four episodes of meningitis in a five-year period associated with chronic intractable migraine and papilledema attributed to Mollaret’s meningitis.

## Introduction

In 1944, French Neurologist Pierre Mollaret identified a rare form of recurrent, benign meningitis, which was named “Mollaret’s meningitis.” It is characterized by episodes of aseptic meningitis that resolve spontaneously with no residual neurological deficit [1]. The rate of recurrence is variable, with episodes ranging in frequency from every few weeks to intervals of many years. The overall course of the disease has been documented to range from three to 28 years [1]. Herpes simplex virus type 2 (HSV-2) is the most commonly isolated pathogen in this condition. The acute phase may produce transient neurological symptoms but no permanent impairment. We describe the case of a female who presented with five episodes of meningitis in a five-year period associated with chronic intractable migraine and papilledema.

## Case presentation

 A 44-year-old female was admitted to the hospital with new-onset headache for one day. She had a very severe, dull, constant frontal headache, which was associated with photophobia, nausea, neck pain with movement, fever, and chills. She had no history of migraine. She had had a spinal steroid injection for back pain a few days prior to presentation. Her past medical history was significant for hypertension, hyperlipidemia, diabetes mellitus type 2, non-occlusive coronary artery disease, hypothyroidism following thyroidectomy, bipolar disorder, chronic back pain due to lumbar disc protrusion, and bilateral upper extremity paresthesia associated with cervical spinal stenosis. Her family history was significant for heart disease, diabetes, hypertension, and seizure disorder, and her personal history included smoking. Physical exam revealed a fever of 102.0°F and positive Kernig’s and Brudzinski signs. She remained alert and oriented. She had leukocytosis of 22700/microL, elevated hemoglobin of 16.6 g/dl, and elevated platelet of 467000/microL. Cerebrospinal fluid (CSF) polymerase chain reaction (PCR) was positive for HSV-2 while the CSF culture was negative (Table 1). She was treated with a 14-day course of intravenous (IV) acyclovir. The repeat CSF analysis in a month showed that the HSV-2 PCR was negative.

**Table 1:** CSF analysis during each episode of aseptic meningitis CSF (cerebrospinal fluid), PCR (polymerase chain reaction), HSV (herpes simplex virus), RBC (red blood cell), N/A (not available)

	First episode	Follow-up in a month	Second episode	Admission for (migraine)	Third episode	Fourth episode
HSV-1 PCR	Negative	Negative	Negative	Negative	Negative	Negative
HSV-2 PCR	Positive	Negative	Positive	Negative	Positive	Negative
Nucleated cells (N=0-5 /CUMM)	215	33	1268	0	230	3
RBC (N=0/CUMM)	6	8	6	1	20	11
Neutrophils (N=0-6 %)	7	0	18	N/A	4	N/A
Lymphocytes (N=40-80%)	53	94	75	N/A	84	N/A
Monocytes (N=15-45%)	39	6	7	N/A	12	N/A
Total protein (N=15-45 mg/dL)	161	43	262	29	73	32
Glucose (N=40-70 mg/dL)	62	77	76	75	75	102

After the first episode of HSV-2 meningitis, she developed chronic, uncontrolled migraines with staring spells as well as myoclonic jerks related to migraine. Her migraine and myoclonic jerks were managed by valproic acid, gabapentin, sumatriptan, and oxycodone as needed, and botulinum toxin (Botox) injections every six weeks. She had a mild improvement of migraine and myoclonic jerks with this treatment over the next three years. During this time period, however, she was diagnosed with depression and post-traumatic stress disorder (PTSD) and treated with sertraline and venlafaxine.

Three years after the first episode, she had meningitis again, with a similar clinical presentation as before, with fever, headache, and positive Kernig’s and Brudzinski signs. The CSF study showed elevated opening pressure (OP) of 37 cmH2O and positive HSV-2 PCR (Table 1). She underwent treatment with IV acyclovir for 10 days, followed by oral valacyclovir for two weeks as prophylaxis. Electroencephalography (EEG) did not show any epileptiform activities. Head imaging was negative for any abnormalities. After the second episode of HSV-2 meningitis, her migraine symptoms had worsened in intensity, frequency, and duration, and she developed dizziness, vertigo, and transient hearing loss. Despite being on higher doses of gabapentin, valproic acid, sertraline, and venlafaxine, her migraine was not controlled and she resumed Botox injections, which were effective.

A year later, she was admitted for a severe intractable migraine attack with negative CSF, HSV, PCR, and CSF leukocytosis (Table 1). She did not have fever or neck stiffness. Upon discharge, she was started on topiramate for migraine prophylaxis. She subsequently developed an intermittent pulsatile headache with ptosis, blurred/double vision, and tinnitus. There was a concern for pseudotumor cerebri. Repeat lumbar puncture (LP) was done as an outpatient and showed normal OP - 13 cm of water and normal closing pressure (CP) - less than 10 cm of water. She had a dilated fundus exam, which showed papilledema without vision disturbances. Weight loss along with Botox injections somewhat helped with her headaches.

Eight months later, she had the third occurrence of aseptic meningitis with similar presentation as the previous episodes with fever, headache, and positive Kernig’s and Brudzinski signs. LP showed OP - 22 cm of water and CP - 13 cm of water. CSF, HSV-2, and PCR were positive (Table 1) and she received treatment with IV acyclovir for two weeks. Her headache slightly improved upon completion of acyclovir while she continued other medications as previously described.

Again, eight months later, the fourth episode of aseptic meningitis occurred, which was negative for virus etiology (Table 1). She presented with a severe, throbbing headache, fever, and positive Kernig’s and Brudzinski sign. Her symptoms improved with supportive treatment within three days. She continues to manage the intractable migraines with analgesics and Botox.

## Discussion

Mollaret’s meningitis is known for being recurrent but benign, with no long-term complications. Diagnosis is typically made after three episodes of aseptic meningitis with the characteristic pattern of the total resolution of symptoms between episodes. Mollaret’s can present with transient neurological deficits but no prolonged or long-term residual complication. Our patient developed chronic intractable migraine with aura after the first episode of aseptic meningitis. Her migraine got worse with each episode of meningitis, causing dependence on multiple medications, psychological stress in the setting of constant pain, and multiple hospitalizations.

In 1981, Bruce H. Dobkin discussed that aseptic meningitis can produce a secondary migraine by causing the vasomotor instability of meningeal circulation by the inflammation of small, penetrating meningeal vessels to the cortex [2]. This mechanism of developing a migraine could be applied to this case. Our patient also developed papilledema, which is defined as edema of the optic disc as the result of elevated intracranial pressure (ICP). Elevated ICP can present without papilledema; however, papilledema cannot be present without elevated ICP as per the definition of papilledema [3-4]. However, papilledema is extremely rare and transient in aseptic meningitis and most of these cases have normal ICP. The mechanism of development of papilledema in aseptic meningitis is likely due to pleocytosis and meningeal inflammation rather than elevated ICP [4]. ICP can transiently increase in acute meningitis, which eventually normalizes. Our patient has developed papilledema with normal ICP after the third episode as the result of recurrent aseptic meningitis. However, subsequent episodes were not investigated for papilledema. In this very rare presentation of Mollaret’s meningitis, chronic meningeal inflammation may have caused papilledema and migraine. Our patient also developed myoclonic jerk, ptosis, and tinnitus.

Recurrent episodes of meningitis can lead to intractable migraine with increasing severity with each episode and can produce resistance to analgesics. Over time, the patient might require a higher dose of opioids. Topiramate and Botox injections helped with migraine prophylaxis and treatment by decreasing the need for a higher dose of opioids and other analgesics. In our case, Botox was proven more beneficial for controlling symptoms.

Etiology

This patient had aseptic meningitis five times in a five-year period, three of which were associated with HSV-2. This is consistent with HSV-2 being the most commonly isolated pathogen in Mollaret’s meningitis (up to 85% of cases) [5]. Mollaret’s meningitis is more common in females than in males [5]. Various etiologies have been associated with Mollaret’s meningitis, including spinal injections, minor head trauma, postpartum pituitary necrosis, low serum immunoglobulin G (IgG), family association, and familial Mediterranean fever [1,6-10].

While HSV-2 is considered the major underlying etiology of Mollaret syndrome, patients do not necessarily have concurrent genital lesions, though some patients always present with genital ulcers before the onset of CNS symptoms [11].

Diagnosis and pathophysiology

Clinical presentation is similar to bacterial meningitis with headache, fever, and positive meningeal signs. A patient can have a transient neurological disturbance, such as seizures, hallucinations, diplopia, cranial nerve paresis, abnormal reflexes, or coma, but no permanent neurological sequel [1,5,9]. Characteristic features and diagnostic criteria (by Bruyn et al. in 1962) of Mollaret’s meningitis are described in Tables 2-3.

**Table 2:** Characteristic features of Mollaret’s meningitis Note: Data from Galdi, Farazmand, Woolley, Kinghorn, Abou-Foul, Buhary, Gayed SL [4-5,11]

1	Recurrent episodes of aseptic meningitis
2	Symptoms free interval between the episodes
3	Spontaneous remission of symptoms
4	Transient neurological symptoms in 50% of the cases
5	No permanent neurological sequelae
6	The probable causative agent is Herpes Simplex Type 2 virus
7	Fever (not always present)
8	Genital symptoms absent in at least half of the cases
9	There may be increased gamma globulin fraction in the CSF

**Table 3:** Diagnostic criteria for Mollaret’s meningitis Note: Piskin IE, Yarimay G. Mollaret meningitis [2]

1	Recurrent episodes of severe headaches, meningismus, and fever.
2	CSF pleocytosis of lymphocytes, neutrophils, and endothelial cells
3	Symptom-free resolution of symptoms and signs during the episodes
4	Spontaneous resolution of symptoms and signs during the episodes
5	Absence of detectable etiology

Leukocytosis is usually present. Leukopenia and eosinophilia have been noted previously [1]. Imaging usually remains negative [1,12]. CSF analysis can show clear CSF, pleocytosis, lymphocytosis, unusual large lymphocytes or mononuclear cells, and endothelial cells (Mollaret cells) [1]. Elevated neutrophils in CSF have been noted in some cases [12]. There will be elevated protein >100 and normal glucose. CSF gamma globulin, IgG level, and oligoclonal IgG bands have been elevated in past cases [1,4,8,13]. Culture usually remains negative.

Mollaret cells are described as large monocytic cells with nuclei shaped like a bean, footprint, or cloverleaf, and deep nuclear clefts [14-15]. Large, degenerated monocytes that appear as “ghost cells” can be present as well [14]. These cells are highly fragile and difficult to see after 24 hours from the onset of the attack and require a special stain (Papanicolaou). Mollaret cells are formed when mononuclear cells have been invaded by a virus and have been seen in varicella-zoster virus meningitis, Sjogren’s disease, neuro-Behcet disease, sarcoidosis, and Vogt-Koyanagi-Harada disease [4]. CSF analysis has shown a rise in the IgG index and constant oligoclonal bands in some cases [1,13].

Treatment

Mollaret’s meningitis is a self-limiting and benign disease and should be treated with supportive measures. It is often treated with seven to 14 days of IV acyclovir. Treatment with acyclovir or valacyclovir is controversial. A randomized control trial of valacyclovir as suppressive therapy for recurrent aseptic meningitis showed no prevention of recurrent meningitis, and it was not recommended in the treatment of HSV meningitis [16]. There has been debate on the utility of prophylactic oral acyclovir, famciclovir, or valacyclovir for patients with this condition. Some case reports have demonstrated that valacyclovir has provided long-term relief, though other studies have not indicated a significant impact from prophylaxis [11,16]. Colchicine has generally failed as prophylaxis, but some cases had the benefit of meningitis-free years [10,12,17]. One case of recurrent aseptic lymphocytic meningitis has been documented in the setting of familial Mediterranean fever and responded to prophylactic colchicine treatment [7]. One case report of recurrent HSV-2 genital ulcers is correlated with recurrent meningitis episodes in which chronic suppressive treatment with acyclovir 800 mg daily showed significant improvement [11].

Prevalence of conditions

Table 4 lists the various conditions associated with Mollaret’s meningitis based on our literature search.

**Table 4:** Conditions associated with Mollaret’s meningitis HSV (herpes simplex virus), EBV (Epstein-Barr virus), NSAIDs (non-steroidal anti-inflammatory drugs), OKT (Orthoclone OKT3{muromonab-CD3}) Note: Data from [1-2,4,7-12,14-15,18-20]

1	HSV-2 (most common)
2	HSV-1
3	Varicella-Zoster virus
4	HSV-6
5	Dermoid or epidermoid cyst
6	HSV-2 genital ulcers
7	Enterovirus infection
8	Migraine syndrome
9	Familial Mediterranean fever
10	Intracranial or intraspinal cyst
11	EBV infection
12	Behcet’s disease
13	Sarcoidosis
14	Systemic lupus erythematosus
15	Intracranial tumor
16	Drug-induced (penicillin, cephalosporin trimethoprim-sulfamethoxazole, NSAIDs, immune globin intravenous, OKT3)
17	Vogt-Koyanagi-Harada syndrome
18	Whipple disease
19	Allergic disease
20	Postpartum pituitary necrosis

## Conclusions

It is important to diagnose this syndrome early to reduce tests and antibiotic use, but this rare meningitis with subsequent migraine makes the diagnosis challenging. Mollaret’s is a benign syndrome with a good prognosis, but our case is unusual with severe migraine as a complication. Important considerations include a dilated fundus exam to look for papilledema and a Botox injection as treatment for chronic intractable migraine. Post-Mollaret’s migraine should be differentiated from another attack of Mollaret’s meningitis and symptoms should be treated adequately.
